# Esophagopericardial fistula as a rare complication after total gastrectomy for cancer

**DOI:** 10.1186/1477-7819-7-58

**Published:** 2009-07-06

**Authors:** Nikolaos Dafnios, Georgios Anastasopoulos, Athanasios Marinis, Andreas Polydorou, Georgios Gkiokas, Georgios Fragulidis, Panayiotis Athanasopoulos, Theodosios Theodosopoulos

**Affiliations:** 1 Second Department of Surgery, Areteion University Hospital, Athens Medical School, National and Kapodistrian University of Athens, 76 Vassilisis Sofia's Ave, 11528, Athens, Greece

## Abstract

**Background:**

Esophagopericardial fistula is a rare but life-threatening complication of benign, malignant or traumatic esophageal disease. It is most commonly associated with benign etiology and carries a high mortality rate which increases with delay in diagnosis.

**Case presentation:**

We present a case of an esophagopericardial fistula as a rare complication in a 53-year-old male patient, 7 months after total gastrectomy for an adenocarcinoma of the esophagogastric junction.

**Conclusion:**

The prognosis of esophagopericardial fistula is poor, especially when it is associated with malignancy.

## Background

Esophagopericardial fistula (EPF) is a rare clinical entity which carries a dismal prognosis and is associated with benign, malignant or traumatic disease of the esophagus. Esophageal ulcers, chronic esophagitis, foreign body impaction, post-bouginage perforation and breakdown of anastomotic sites are the most common benign causes. Clinical symptoms include retrosternal pain, dyspnea and fever. Pneumopericardium is the most common radiographic finding, while upper GI series may demonstrate the fistulous tract or the accumulation of the contrast material inside the pericardial sac. Endoscopy may reveal the orifice of the fistulous tract or evidence of the underlying pathology. In this report we present a case of an EPF as a rare complication after total gastrectomy for gastric cancer. The prognosis of EPF is poor, especially when it is associated with malignancy.

## Case presentation

A 53-year-old male patient underwent a total gastrectomy for an adenocarcinoma of the esophagogastric junction with an esophagojejunal reconstruction in Roux-en-Y configuration. Histology of the surgical specimen showed a moderately differentiated adenocarcinoma of the esophagogastric junction, with a maximal diameter 5 cm, microscopically positive proximal margins and 21 negative lymph nodes (T3, N0, M0). The patient developed postoperatively a leakage from the esophagojejunal anastomosis, which was treated endoscopically with placement of a covered stent. Post-discharge, the patient received adjuvant radio- and chemo-therapy.

Several months after surgery the patient was re-admitted due to progressive dyspnea, retrosternal pain and hypotension. Physical examination revealed a dyspneic patient with dilated jugular veins and a remarkable diminution of respiratory sounds on the left side, diminished heart sounds and a two-component friction rub during thorax auscultation. Vital signs included a low systolic blood pressure (75 mmHg), tachycardia (120 bpm), tachypnea (35 breaths per minute) and normal body temperature, without significant changes in the electrocardiograph. Laboratory studies revealed a normal hemoglobin (12 g/dl) and elevated white blood cells (26.000/mm^3^), creatinine kinase (720 U/L) and LDH (530 U/L), with negative troponine-I. Chest radiograph demonstrated a moderate left pleural effusion and subsequent pleurocentesis was performed with aspiration of about 2,4L of serosanguineous fluid. Biochemical analysis of the fluid revealed glucose 246 mg/dl, proteins 2,7 g/dl, albumin 1,7 g/dl and LDH 125 U/l, while cytological examination was suspicious for malignant cells. The patient exhibited a moderate amelioration of his symptoms just after the pleurocentesis, but remained hemodynamically unstable and was transferred to the surgical intensive care unit (SICU) for further monitoring.

**Figure 1:**
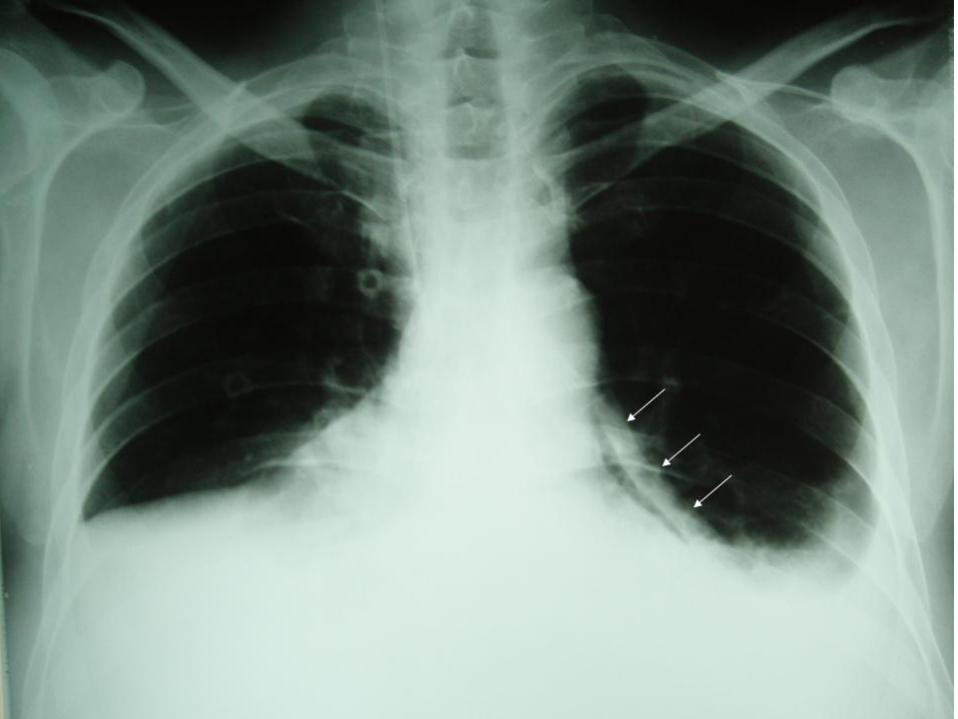
**Plain chest radiograph demonstrating the presence of air in the left lateral pericardium (arrows) along with a small left pleural effusion**.

**Figure 2:**
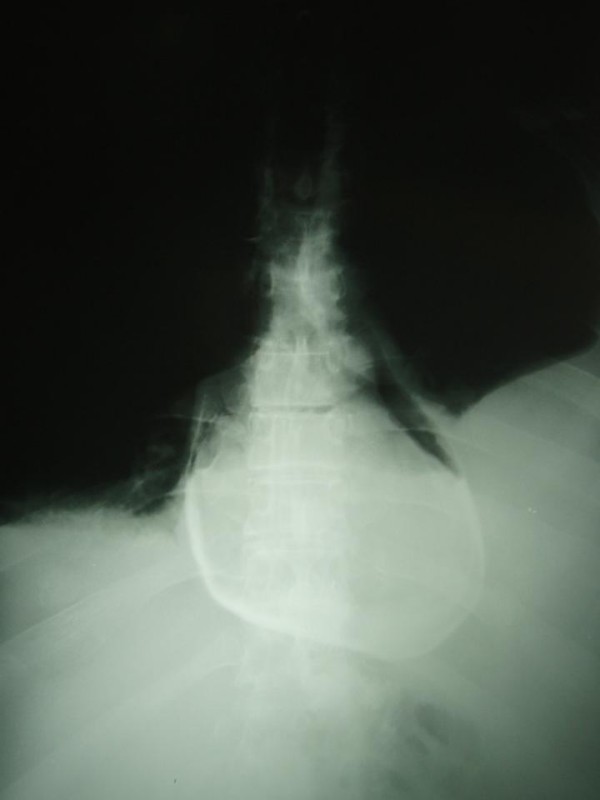
**Filling of the pericardial sac after orally administered water-soluble contrast medium (Gastrographin^®^)**.

**Figure 3:**
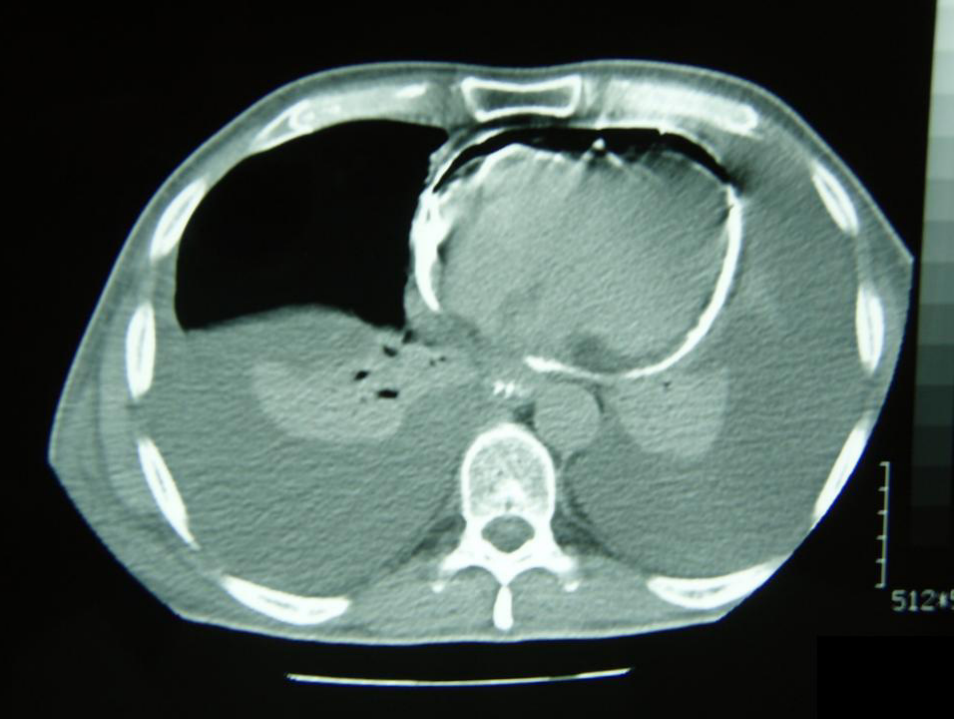
**Thoracic computed tomography scan demonstrating hydropneumopericardium (air and contrast material filling the pericardial sac) and bilateral pleural effusions**.

A new chest radiograph two hours later demonstrated pneumopericardium along the left heart border (Figure 1). Echocardiography revealed air and small pericardial fluid collection, not adequate for pericardiocentesis. The patient eventually stabilized hemodynamically six hours after his admission to the SICU. Upper GI series using water-soluble contrast (Gastrografin^®^) were performed the next day and demonstrated leakage of the contrast from the esophagus and entrance in the pericardium (Figure 2), while thoracic computed tomography (CT) showed hydropneumopericardium (Figure 3), findings suggestive of an esophagopericardial fistula. However, rapid re-accumulation of fluid in the left hemithorax necessitated the placement of a thoracic tube, with a daily output of about 1,5 L serosanguineous fluid. Cytology was positive for malignancy and a pleurodesis was performed. Unfortunately, the patient deteriorated clinically during the following 5 weeks and finally died. Permission for postmortem examination was denied.

## Discussion

Esophagopericardial fistula is a rare and usually life-threatening complication of benign, malignant or traumatic esophageal disease. Benign esophageal disease is by far the most common cause of EPF, accounting for 76% of the cases, while malignancy accounts for only 24% of all reported cases [1-14]. In some of these cases the esophageal cancer was associated with achalasia [12,13]. About one-third (35%) of all cases are due to either esophageal ulceration or chronic esophagitis, often associated with hiatus hernia, reflux and stricture. Perforation by an ingested foreign body is the second most common benign etiology, which occurs in 16% of the cases [15]. The usual site of foreign body impaction is the upper esophagus, just below the cricopharyngeal junction [9]. Iatrogenic causes, such as post-bouginage perforation and anastomotic disruption, account for 6% of all cases of EPF [3,10,11]. Tuberculous abscess formation was at one time a relatively common cause of EPF, but is rarely seen today.

Clinical findings highly suggestive of EPF include retrosternal pain, fever, dyspnea and the presence of a water-wheel murmur [16]. However these clinical manifestations vary and may be overshadowed by major life-threatening complications of pericardial infection [14,5,17]. This emphasizes the central role of radiographic studies in establishing diagnosis.

Pneumopericardium is the most common radiographic finding, present in 50% of the cases and often seen along the left border in the chest radiograph [15], as in our case. Pleural effusions usually on the left hemithorax and pulmonary infiltrates are present in 20% of cases [15]. Once pneumopericardium is recognized, both esophagographic and esophagoscopic studies should be performed to demonstrate a possible fistula. Either a fistulous tract is identified or there is gross filling of the pericardial sac with contrast material in 80% of the cases, on upper GI contrast studies. In our case, no fistulous tract was demonstrated, but hydropneumopericardium and filling of the pericardial sac with contrast material were obvious. Endoscopic studies may reveal such fistulae, as well as the underlying pathology. Echocardiography may demonstrate hydropneumopericardium and can estimate the cardiac tamponade effect. In our case we performed echocardiography just after the evacuation of the left hemithorax in order to assess the pericardial collection.

EPF carries a high mortality rate which increases with delay in diagnosis [6]. Because of the rarity of this clinical entity, little can be learned regarding therapy. Early diagnosis and treatment, including pericardial drainage and intense antibiotic therapy, followed by a well-planned operative closure of the fistula are of paramount importance for the successful management of EPF. Although a successful management of EPF complicating esophagogastrectomy by a modification of Abboo's T-tube technique, together with a pericardial window, multiple drainage tubes, systemic antibiotics and hyperalimentation have been described [18], in our case we preferred a more conservative management due to the rapid resolution of the signs of cardiac tamponade and the documentation of disseminated malignancy.

Although the treatment of an esophagopericardial fistula using an esophageal stent has been widely described [19-21], the potential causative role of the stent in the development of an EPF has not been definitively established. On the other hand, anastomotic leakage has been certainly associated with the development of EPF [18].

Finally, although positive surgical margins after resection of esophageal cancer could be assumed to have a potential role to the development of an EPF, lacking evidences from the literature, however, cannot let us draw any definite conclusions.

## Consent

Written informed consent was obtained from a relative of the patient for publication of this case report and any accompanying images. A copy of the written consent is available for review by the Editor-in-Chief of this journal.

## Competing interests

The authors declare that they have no competing interests.

## Authors' contributions

GA, AM and PA designed and drafted the manuscript; GP, AP and GG critically revised the manuscript; TT and ND finally approved the manuscript and images submitted.

## References

[B1] Gellman DD, Silberstein K (1956). Perforation of peptic ulcer of the oesophagus into the pericardial cavity: report of a case. Br Med J.

[B2] McDaniel JR, Knepper PA (1957). Esophagopericardial fistula: report of a case and review of the literature. J Thorac Surg.

[B3] Praüer HW (1976). [Esophagopericardial fistula with tension pneumopericardium]. Chirurg.

[B4] Price D, Perkes E, Farman J (1980). Pericardial complications of peptic ulceration. Gastrointest Radiol.

[B5] Curry N, Anderson RS (1974). Pneumopericardium and esophago-pericardial fistula following chronic esophagitis presenting as acute respiratory distress. Chest.

[B6] Miller GE, Berger SM (1974). Letter: Esophagopericardial fistula with survival. JAMA.

[B7] Mansour KA, Teaford H (1973). Atraumatic rupture of the esophagus into the pericardium simulating acute myocardial infarction. A case report. J Thorac Cardiovasc Surg.

[B8] Arens RA, Stewart E (1934). Pneumopericardium following a foreign body in the esophagus. Radiology.

[B9] Peeler MB, Riley HD (1957). Cardiac tamponade due to swallowed foreign body. AMA J Dis Child.

[B10] Herrington JL, Ibramin A (1977). Complications of repeated operations to control esophageal reflux (esophagogastrocutaneous and esophagogastropericardial fistulas). Am Surg.

[B11] Schumacher KA (1979). [Pneumopericardium and contrast medium filling of the pericardium after an esophagoantrostomy]. Rofo.

[B12] Strong RW (1974). Oesophago-cardiac fistula complicating achalasia. Postgrad Med J.

[B13] Kudchadkar A, Markovitz W, Moqtaderi FF, Wilder JR (1980). Esophago-diverticulo-pericardial fitula. N Y State J Med.

[B14] Welch TG, White TR, Leis RP, Altieri PI, Vasko JS, Kilman JW (1972). Esophagopericardial fistula presenting as cardiac tamponade. Chest.

[B15] Cyrlak D, Cohen AJ, Dana ER (1983). Esophagopericardial fistula: causes and radiographic features. AJR Am J Roentgenol.

[B16] Meltzer P, Elkayam U, Parsons K, Gazzaniga A (1983). Esophageal-pericardial fistula presenting as pericarditis. Am Heart J.

[B17] Bozer AY, Saylam A, Ersoy U (1974). Purulent pericarditis due to perforation of esophagus with foreign body. J Thorac Cardiovasc Surg.

[B18] Shahian DM, Kittle CF (1981). Successful management of esophagopericardial fistula complicating esophagogastrectomy. J Thorac Cardiovasc Surg.

[B19] Dy RM, Harmston GE, Brand RE (2000). Treatment of malignant esophagopericardial fistula with expandable metallic stents in the presence of esophageal varices. Am J Gastroenterol.

[B20] Nakshabendi IM, Havaldar S, Nord HJ (2000). Pyopneumopericardium due to an esophagopericardial fistula: treatment with a coated expandable metal stent. Gastrointest Endosc.

[B21] Tukkie R, Hulst RW, Sprangers F, Bartelsman JF (1996). An esophagopericardial fistula successfully treated with an expandable covered metal mesh stent. Gastrointest Endosc.

